# Gastrojejunal Anastomosis Complications and Their Management after Laparoscopic Roux-en-Y Gastric Bypass

**DOI:** 10.1155/2015/698425

**Published:** 2015-10-18

**Authors:** Yannick Fringeli, Marc Worreth, Igor Langer

**Affiliations:** Department of Surgery, Hospital of Jura, Faubourg des Capucins 30, 2800 Delémont, Switzerland

## Abstract

*Background*. Complications at the gastrojejunal anastomosis after laparoscopic Roux-en-Y gastric bypass (LRYGB) are challenging in terms of diagnosis, therapy, and prevention. This study aims at identifying these complications and discussing their management. *Methods*. Data of 228 patients who underwent a LRYGB between October 2008 and December 2011 were reviewed retrospectively to evaluate the frequency and treatment of complications such as stenoses, marginal ulcers, perforated marginal ulcers, or anastomotic leaks related to the operation. *Results*. Follow-up information was available for 209 patients (91.7%) with a median follow-up of 38 months (range 24–62 months). Of these patients 16 patients (7.7%) experienced complications at the gastrojejunostomy. Four patients (1.9%) had stenoses and 12 patients (5.7%) marginal ulcers, one of them with perforation (0.5%). No anastomotic leaks were reported. One case with perforated ulcer and one with recurrent ulcers required surgical revision. *Conclusion*. Gastrojejunal anastomotic complications are frequent and occur within the first few days or up to several years after surgery. Stenoses or marginal ulcers are usually successfully treated nonoperatively. Laparoscopic repair, meanwhile, is an appropriate therapeutic option for perforated ulcers.

## 1. Introduction

In the last decades obesity has dramatically increased and is a new global epidemic. Since 1980, obesity has nearly doubled worldwide and the World Health Organisation reported that 200 million men and nearly 300 million women were obese in 2008 [1]. For severe obesity (body mass index (BMI) ≥ 35 kg/m^2^) and especially for morbid obesity (BMI ≥ 40 kg/m^2^), conservative therapies showed limited results. In contrast, bariatric surgery is currently the most effective and sustainable treatment for weight loss [2]. Moreover, several studies support the positive impact on comorbidities and a decrease in overall mortality after bariatric surgery when compared to conservative treatment [3, 4]. Among the different bariatric procedures, laparoscopic Roux-en-Y gastric bypass (LRYGB) was the most commonly performed operation worldwide for obese patients in 2008 [5]. Although this technique is one of the oldest, it still constitutes the gold standard in the field of obesity surgery. Despite excellent surgical outcome [6], early and late complications after Roux-en-Y gastric bypass remain a challenge in their detection and management for both health professionals and experienced bariatric surgeons. The most frequently and potentially serious complications concern the gastrojejunal anastomosis. Anastomotic stenoses and marginal ulcers are by far the most common complications with incidence rates of 1–28% [2, 7–10] and 0.6–16% [2, 11–13], respectively. They can occur independently or simultaneously. Leaks at the gastrojejunostomy (GJ) and marginal ulcer perforations are rare but impact tremendously the patient's outcome and often require a surgical revision. This study aims at identifying the type and the incidence of complications at the gastrojejunal anastomosis after LRYGB and discussing their management.

## 2. Materials and Methods

**Figure 1:**
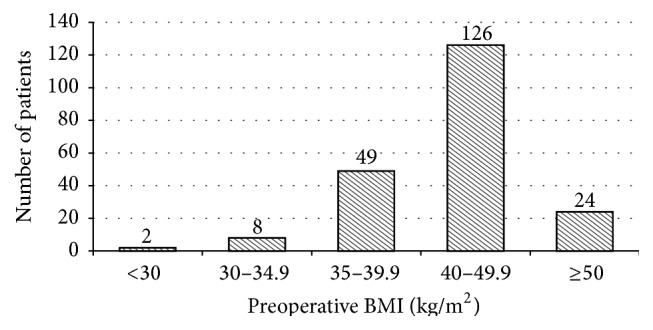
Distribution of patients according to preoperative BMI.

Between October 2008 and December 2011, 228 patients underwent a laparoscopic Roux-en-Y gastric bypass by the same two experienced bariatric surgeons. Of the 228 patients, only patients attending a regular follow-up until January 2014 were included in this study. Data were obtained by retrospective chart analysis, which included demographic data (age, gender, and preoperative BMI), preoperative comorbidities (diabetes mellitus, hypertension, dyslipidemia, obstructive sleep apnea, musculoskeletal disorders, gastroesophageal reflux disease, depression, and smoking), and technical characteristics of the operation (operative time, removal of a gastric banding at the same time, and conversion rate to laparotomy) (see Table 1 and Figure 1). Type, time of occurrence, and treatment of all gastrojejunal complications were subject to analysis. The study also focused on preventable risk factors at the time of complication for patients developing marginal ulcers. These include smoking, alcohol use, and use of nonsteroidal anti-inflammatory drugs (NSAID).

### 2.1. Preoperative Requirements

Preoperative BMI ≥ 40 kg/m^2^ was mandatory for a bariatric procedure. Since November 2010, patients with BMI ≥35 kg/m^2^ could also be included according to the revised guidelines of the Swiss Society for the Study of Morbid Obesity and Metabolic Disorders [14]. Patients with previous implantation of a gastric banding, who presented with band intolerance or band failure requiring a conversion to a gastric bypass, were also included independently of their BMI. All patients underwent an upper endoscopy, which included a screening for* Helicobacter pylori* (HP). HP-positive patients received an adequate HP eradication therapy. A psychological and/or psychiatric assessment was performed for every patient prior to the bariatric procedure with the view to detecting major psychiatric disorders, including alcohol abuse. Patients with chronic alcohol abuse were excluded from the surgery. One month prior to the operation, all patients underwent a high-protein diet to lose weight in order to decrease liver size and to facilitate the operation. The mean weight loss was 5 kg.

### 2.2. Surgical Procedure

Our technique of the LRYGB operation was based on that initially described by Wittgrove [10, 15] and modified with a mechanical antecolic, antegastric end-to-side GJ. In a reverse Trendelenburg position, a 10–15 cm^3^ gastric pouch was created by stapling first horizontally from the lesser curvature and then vertically to the angle of His. An anvil of 21 mm (EEA OrVil, Covidien) was inserted transorally into the pouch fixed on a flexible gastric tube and placed below the first staple line. Approximately 60 cm below the ligament of Treitz, the small bowel was lifted in an antecolic and antegastric direction to the posterior wall of the gastric pouch to perform the end-to-side gastrojejunal anastomosis by using a circular endoluminal stapling technique. Interrupted 3-0 Vicryl sero-serosal sutures were used circumferentially to protect the gastrojejunal anastomosis. Then, a stapled side-to-side jejunojejunal anastomosis was performed to finalize the Roux-en-Y bypass with manual closure of the stapler introduction orifice by using continuous 3-0 Vicryl suture. The length of the alimentary loop was 100 cm for the patients with a preoperative BMI < 50 kg/m^2^ and 150 cm for a preoperative BMI ≥ 50 kg/m^2^. In patients, who already benefited from gastric banding, the band was removed at the beginning of the operation.

### 2.3. Postoperative Management

A gastrografin swallow was performed on the first postoperative day. Patients were then allowed to consume clear liquids and eat small portions of mixed meals under the supervision of a dietician, who provided a detailed diet to pursue after discharge. At discharge, proton pump inhibitors (PPI) therapy and thromboembolic prophylaxis with low-molecular-weight heparin were prescribed for 1 month. All patients were thoroughly informed not to take NSAID and abstain from alcohol. Smoking was also strongly discouraged. Complications were diagnosed by using upper endoscopy only in symptomatic patients who had presented with dysphagia, persistent epigastric pain, nausea, or vomiting and it was not performed routinely.

## 3. Results

**Figure 2:**
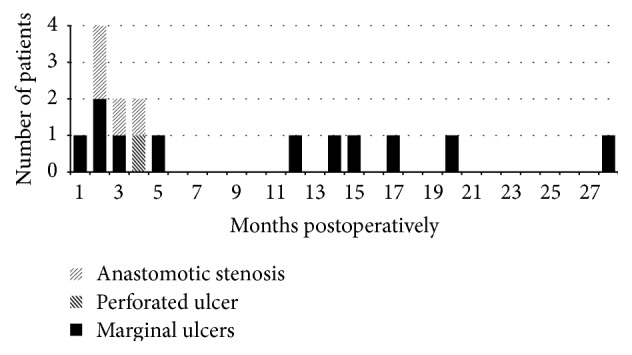
Incidence and type of complications at the gastrojejunal anastomosis over time.

Two hundred nine patients (209/228, 91.7%) attended regular follow-up and were included in this study. The median follow-up was 38 months (range 24–62 months). During this time, a total of 16 patients (16/209, 7.7%) experienced complications at the gastrojejunal anastomosis (see Table 2). Within this group, 4 patients (4/209, 1.9%) suffered from anastomotic stenosis and 12 (12/209, 5.7%) from marginal ulcers, of which one was complicated by a perforation (1/209, 0.5%). The most common symptoms reported were dysphagia (3/209) and epigastric pain (1/209) for patients with stenosis, and epigastric pain (9/209) and bleeding (3/209) for patients with ulcers. No anastomotic leaks were reported. The incidence of the complications over time is shown in Figure 2. Stenoses as postoperative complications occurred within the first 4 postoperative months while ulcer development showed a bimodal distribution with 6 cases (6/12, 50%) occurring within the first 5 months and 6 cases (6/10, 50%) after 1 year.

All cases of anastomotic stenosis were successfully treated with 1–3 repetitive endoscopic dilatations. Ten cases (10/12, 83%) of marginal ulcers were successfully managed conservatively with a PPI therapy as well as cessation of potential risk factors such as smoking, alcohol consumption, and use of NSAID. Among patients who developed marginal ulcer, 9 patients (9/12, 75%) presented with persistent smoking at the time of complication. One of the 9 also presented with concomitant alcohol and NSAID use (1/12, 8.3%), and 2 of the 9 presented with concomitant alcohol (1/12, 8.3%) or NSAID use (1/12, 8.3%).

*Complications Requiring Surgical Therapy.* One case with perforated ulcer and one with recurrent ulcers required surgical revision. The first patient was a 26-year-old woman, with known risk factors of type II diabetes and persistent smoking who presented with symptoms of an acute abdomen and peritonitis 4 months postoperatively. Imaging studies demonstrated free intra-abdominal air and the suspicion of a perforation at the GJ site. Emergency laparoscopy confirmed a perforated ulcer at the gastrojejunal anastomosis with purulent peritonitis. The perforated marginal ulcer was treated laparoscopically with interrupted 3-0 Vicryl suture and omental patch repair. In order to protect the GJ and to facilitate early enteral nutrition, a percutaneous gastrostomy in the bypassed stomach was performed concurrently along with high dose PPI therapy and intravenous antibiotics. The postoperative recovery was uneventful. The percutaneous gastrostomy was removed after 12 days and the patient discharged after 13 days. No stenosis or ulcer recurrence was observed in the follow-up of this patient.

The second patient requiring an operative treatment was a 44-year-old woman, with known risk factors of gastroesophageal reflux disease and persistent smoking. Five years before the LRYGB the patient underwent a gastric banding operation. Twenty-eight months after LRYGB she developed a marginal ulcer of the GJ. The anastomotic ulcer was initially treated conservatively with PPI therapy. The upper endoscopic control 3 months later confirmed good healing of the lesion and ruled out stenosis. Subsequently burning epigastric pain with dysphagia and vomiting reoccurred. The endoscopic control showed a recurrence of the ulcer of the GJ, which was resistant to conservative treatment and required an open resection of the anastomosis and a new Roux-en-Y reconstruction of the GJ 37 months after the initial operation. The latter operation resulted in abdominal sepsis due to an infected hematoma and required repetitive revisions with peritoneal lavage and open treatment with a vacuum-assisted closure system in combination with implantation of a Vicryl mesh in inlay technique.

## 4. Discussion

This report describes a complication rate of 1.9% for anastomotic stenoses, 5.7% for marginal ulcers, and 0.5% for perforated ulcers at a median time of 38 months after LRYGB. These findings corroborate the rate of complications reported in previous studies that describe a stenosis rate of 1–28% [7, 8, 10], 0.6–16% for marginal ulcers [11–13], and 0.4–1% for perforated ulcers [16–19] after Roux-en-Y gastric bypass. The wide range of these results reflects a diversity of research protocols, different surgical techniques used, and whether the study was performed only in symptomatic patients or as a routine control in all patients. The real rates of these complications are therefore difficult to assess accurately and are probably often underestimated.

In our study, anastomotic stenoses occurred all within the first 4 months after LRYGB. Stenoses at the gastrojejunal anastomosis are one of the most frequent early complications after gastric bypass [20]. It typically appears 3–6 weeks after the operation [21] and is followed by such symptoms as dysphagia, nausea, vomiting, and gastroesophageal reflux. The etiology remains uncertain, but it seemingly depends on local factors (ischemia, scar formation, and tension of the anastomosis) and on the technique used to create the GJ (i.e., handsaw, circular versus linear stapler, and size of the stapler). For instance, Nguyen et al. reported a higher rate of stenoses using a 21 mm (26.8%) compared to a 25 mm (8.8%) circular stapler without compromising weight loss [22]. Similarly in our report, anastomotic stenoses can usually be treated safely and effectively with endoscopic dilatation [20, 22, 23]. Occasionally, gradual dilatation over repetitive sessions is necessary and may reduce the risk of perforation [7].

Further complications revealed in our study are marginal ulcers. Several previous studies attempted to define potential risk factors in the development of marginal ulcers, but it still remains a controversial topic. It is particularly true as regards the exact role of HP [13, 24] confounded by other possible causes reported in the literature. There is currently no evidence for an association between the development of marginal ulceration after LRYGB and the presence of an ongoing HP infection. HP appears rather to cause an injury to the gastric mucosa preoperatively that potentiates the formation of marginal ulcer after gastric bypass [13]. Therefore HP eradication is recommended in all positive patients prior to surgery. Other causes range from smoking, alcohol consumption, use of NSAID, diabetes, excess acid exposure due to creation of a too large gastric pouch, to a dilatation of the gastric pouch over time, or to the presence of a gastrogastric fistula, presence of foreign body such as nonabsorbable sutures or staples, local factors such as ischemia, or tension at the GJ [7, 13, 24, 25]. In our study 75% of patients developing marginal ulcers were persistent smokers at the time of diagnosis. This percentage is more than double the prevalence of smokers among all patients before LRYGB (72/209, 34.4%). This finding strengths the importance to encourage patients to stop smoking before and, first of all, after LRYGB. Counselling, encouragement and nicotine substitutes (e.g., transdermal patches and gums) are possible options to help patients stop smoking. Alcohol and NSAID use were each found in 2 patients (2/12, 16.7%) concomitantly with smoking. Even if their exact role in the pathogenesis of marginal ulcers after LRYGB is not yet clearly understood, both factors are believed to predispose patients to marginal ulcers and have to be avoided.

The apparition of marginal ulcers over time showed a bimodal distribution with 6 patients (6/12, 50%) developing marginal ulcers within the first 5 months and 6 patients (6/12, 50%) after 1 year. This reflects the formation of* early* and* late* marginal ulcers as described in former studies [12, 26, 27]. Even if marginal ulcers are multifactorial, the development of* early* marginal ulcer is more likely associated with local factors (ischemia, postoperative inflammation, stenosis, and foreign body) while* late* marginal ulcers are likely to be related to an increased acid exposition of the GJ developing over time [11, 26]. For both types of ulcers, the treatment is identical and consists of a minimum of 3- to 6-month PPI therapy, elimination of potential risks factors, and regular endoscopic control to monitor healing and rule out stenosis. The conservative treatment is especially long for* late* marginal ulcers with a mean healing time of 7 months described by Csendes et al. [26]. Recurrent marginal ulcers refractory to medical therapy are often due to local problems such as enlargement of the gastric pouch over time or presence of a gastrogastric fistula with subsequent increased acid exposure of the jejunal mucosa. The presence of a Zollinger-Ellison syndrome has also to be ruled out in these patients. As we experienced with one patient, intractable marginal ulcers after gastric bypass oblige to perform revisional surgery. The operation consists of a resection and reconstruction of the gastrojejunal anastomosis with or without partial remnant gastrectomy.

Perforated marginal ulcer is another serious and potentially life-threatening complication following LRYGB. In our study, we detected a perforated ulcer in one patient 4 months postoperatively, which was efficiently treated with a laparoscopic suture repair followed by reinforcement using an omental patch. In their review of 3,430 procedures of LRYGB, Felix et al. identified 35 cases of perforation (1%) with a median time to perforation of 18 months (range 3–70 months) [17]. Wendling et al. have recently described the most delayed onset of perforated ulcer found in the literature, occurring 98 months after original surgery [19]. Predisposing factors for perforated ulcers are likely the same as the above mentioned for marginal ulcers. In the study of Felix et al., incidence of smoking was significantly higher and the use of NSAID and steroids were commonly found in patients presenting with a perforated ulcer [17]. The use of NSAID was reported in 6 of the 7 cases of perforated ulcers in the study of Sasse et al. [16]. In our study, only smoking was found to be highly associated with the development of marginal ulcers. To date, there is no consensus regarding the optimal therapy for perforated ulcer after gastric bypass. Case series are rare in the literature and include only very small sample sizes. The laparoscopic repair using an omental patch appears nevertheless to be a safe and effective therapeutic option [18, 19, 28]. It needs however to be performed by surgeons familiar with minimally invasive technique in bariatric surgery.

Some limitations of this study have to be acknowledged. First, this study was retrospective, with incomplete data on all possible risk factors and on their evolution over time. Due to a small number of complications, it was difficult to consistently determine predisposing factors leading to the development of GJ complications.

## 5. Conclusion

Complications at the gastrojejunal anastomosis after LRYGB are frequent and potentially life-threatening. They appear in the first few days following surgery or several years after the initial operation. Symptoms such as dysphagia, persistent epigastric pain, nausea, or vomiting must be investigated early on and patients have to be referred to a bariatric specialist. The upper endoscopy plays a key role in the diagnosis. In most cases stenoses or marginal ulcers are successfully treated nonoperatively while perforated ulcers require urgent surgical repair with laparoscopy being the most feasible choice. Close follow-up and suppression of potential risk factors, especially smoking, alcohol consumption, use of NSAID, or steroids are key factors in the reduction of complications at the GJ and must be discussed with the patient already preoperatively.

## Figures and Tables

**Table 1:** Demographics, comorbidities, and operative and postoperative characteristics.

	Patients (*n* = 209)
*Demographics*	
Gender (women)	159 (76.1%)
Median age (years)	41 [15–67]
Median BMI (kg/m^2^)	43 [27–61]
*Comorbidities*	
Diabetes	53 (25.4%)
Insulin-requiring diabetes	24 (11.5%)
Hypertension	83 (39.7%)
Dyslipidemia	46 (22.0%)
Obstructive sleep apnea	51 (24.4%)
Osteoarticular disorders	130 (62.2%)
GERD	74 (35.4%)
Depression	77 (36.8%)
Tobacco use	72 (34.4%)
*Operative characteristics*	
Median operative time (min)	155 [80–310]
Removal of gastric banding	30 (14.4%)
Conversion to laparotomy	3 (1.4%)
*Postoperative characteristics*	
Median hospital stay (days)	5 [3–180]

BMI = body mass index; GERD = gastroesophageal reflux disease.

**Table 2:** Patient data at the time of complication.

Case number (*n* = 16)	Sex	Age	Type of complication	Predominant symptom	Interval after LRYGB (months)	Therapy	Risk factors at the time of complication (presence +, absence −)		
							Smoking	Alcohol use	NSAID use
1	F	40	Marginal ulcer	GI bleed	0	C	+	−	−
2	M	24	Marginal ulcer	Pain	2	C	+	+	−
3	F	65	Marginal ulcer	GI bleed	2	C	−	−	−
4	F	42	Marginal ulcer	Pain	3	C	+	−	−
5	F	26	Perforating ulcer	Pain	4	S	+	−	−
6	M	30	Marginal ulcer	Pain	5	C	+	+	+
7	F	28	Marginal ulcer	GI bleed	12	C	+	−	−
8	F	40	Marginal ulcer	Pain	14	C	+	−	+
9	F	40	Marginal ulcer	Pain	15	C	−	−	−
10	F	30	Marginal ulcer	Pain	17	C	+	−	−
11	F	41	Marginal ulcer	Pain	20	C	−	−	−
12	F	44	Recurrent ulcers	Pain	28	S	+	−	−

13	F	40	Stenosis	Dysphagia	2	C			
14	F	43	Stenosis	Pain	2	C			
15	M	54	Stenosis	Dysphagia	3	C			
16	M	39	Stenosis	Dysphagia	4	C			

GI = gastrointestinal; C = conservative therapy; S = surgical therapy.
